# Key Targets and Molecular Mechanisms of Active Volatile Components of *Rabdosia rubescens* in Gastric Cancer Cells

**DOI:** 10.2174/1573409918666221003091312

**Published:** 2022-12-14

**Authors:** Yanhui Hu, Qingli Cui, Dongyang Ma, Wenwen Jin, Yingyue Li, Jianhua Zhang, Youqi Xu

**Affiliations:** 1 Department of Integrated Traditional Chinese and Western Medicine, The Affiliated Cancer Hospital of Zhengzhou University & Henan Cancer Hospital, Zhengzhou, Henan 450008, China;; 2 Medical Engineering Technology and Data Mining Institute, Zhengzhou University, Zhengzhou, Henan, 450001, China;; 3 The Second Affiliated Hospital of Nanjing University of Chinese Medicine, Nanjing University of Chinese Medicine, Nanjing, Jiangsu, 210000, China

**Keywords:** *Rabdosia rubescens*, network pharmacology, gastric cancer, signaling pathway, targets, TNF

## Abstract

**Objective:**

To examine the effect and mechanism of volatile components of *Rabdosia rubescens* on gastric cancer.

**Methods:**

Gas chromatography-mass spectrometry was used to detect and identify the volatile components of *R. rubescens*. The network pharmacology method was used to analyze the targets of volatile components of *R. rubescens* in gastric cancer and to reveal their molecular mechanisms. The effects of volatile components of *R. rubescens* on gastric cancer cells were verified by biological experiments.

**Results:**

Thirteen volatile components of *R.rubescens* were selected as pharmacologically active components. The 13 active components had 83 targets in gastric cancer, and a Traditional Chinese Medicine-component-targets gastric cancer network was successfully constructed. Five core targets were obtained: TNF, IL1B, MMP9, PTGS2 and CECL8. The volatile components inhibited the proliferation of gastric cancer cells in a concentration-dependent manner and promoted the apoptosis of gastric cancer cells. The volatile components reduced the levels of TNF, IL1B, MPP9, and PTGS2 in a concentration-dependent manner.

**Conclusion:**

Our study demonstrates the effects of volatile components in *R.rubescens* on gastric cancer and provides preliminary findings on their mechanisms of action.

## 1 INTRODUCTION

Gastric cancer is a malignant tumor caused by pathological changes of gastric mucosal epithelial cells [1, 2]. Traditional Chinese medicine (TCM) has been used for the treatment of various diseases, including malignant tumors. Chinese herbal medicine has been used for cancer treatment and has the advantages of low toxicity and few side effects. Various compounds present in Chinese herbal medicine have anti-tumor effects [3].

*R. rubescens* has been used for the treatment of cancers of the digestive system for more than 30 years. Pharmacological studies have demonstrated that *R. rubescens* has numerous effects, including anti-tumor, anti-inflammatory, anti-bacterial, and immunity enhancing effects [4, 5]. *R. rubescens* is commonly used to treat advanced esophageal cancer, cardiac cancer, and gastric cancer, with both esophageal relaxation and anti-inflammatory effects that can help food ingestion and inhibit the proliferation of tumor cells. These effects can improve symptoms in advanced patients and prolong life. Patients who continue treatment with *R. rubescens* after surgery and/or radiotherapy show reduced recurrence and metastasis. *R. rubescens* can be taken as a single treatment or in combination therapy with other drugs [6]. Interest in the anti-tumor effects of volatile components in TCM has increased in recent years, including the effects of volatile components from *R. rubescens* on gastric cancer.

Network pharmacology, together with computer simulation analysis, bioinformatics, and molecular network data, reveals the mechanism of drug action from different perspectives and is an indispensable part of the efficacy evaluation system [7, 8]. In this study, we used a tumor database and network pharmacology to study the pharmacological action and molecular mechanisms of the volatile components of *R. rubescens* in gastric cancer, with the aim of identifying effective components of TCM for the treatment of gastric cancer. Our findings provide theoretical support for the application of TCM volatile components for cancer treatment.

## 2 MATERIALS AND METHODS

### 2.1 Preparation of Concentrated Volatile Component Extract from *R. rubescens*

The volatile components of *R. rubescens* were extracted by hot water extraction of ethanol, hot water extraction of methanol, and hot water extraction of benzenol combined with rotary distillation.

A 50-g sample of *R. rubescens* (Shenyang Wanlei Biology, Shenyang, China) was weighed and passed through a 60-mesh sieve after ultrafine grinding. Next, three portions of *R. rubescens* powder (10 g) were mixed with liquid 1:50, 500 mL ethanol, benzenol and methanol solutions. After distillation at 67°C, 75°C and 81°C for 4 h, ultrafiltration was used to extract the solvent from the three extracts, and rotary evaporation was used to concentrate the extraction solution to 5 ml. After 0.22 µm oily filter film, a 0.5-ml sample was put into a 1-ml injection bottle for a standby test.

### 2.2 Detection of Volatile Components of *R. rubescens* by Gas Chromatography-mass Spectrometry (GC-MS)

Gas chromatographic conditions were as follows: the chromatographic column was an RTX-5 ms (30 m × 0.25 mm, 0.25 µm) elastic quartz capillary column. The temperature was programmed, and the determination time was 20 min; the shunting ratio was 20:1, the inlet temperature was 280 ^o^C, and the column pressure was 50 KPa.

Mass spectrometry conditions were as follows: EI ion source; the ion source temperature was 200°C; the electron energy was 70 eV; the connector temperature was 250°C; the solvent delay was 3.5 min; the scanning range was 50–600 amu; and the voltage of the electron multiplier was 1200 V.

### 2.3 Screening and Identification of Volatile Components of *R. rubescens*

The TCMSP (Traditional Chinese Medicine Systems Pharmacology Database and Analysis Platform) database was used to screen the volatile components of *R. rubescens*, and the PubChem Compound database, including Canonical SMILES and InChI, was used to obtain the chemical structures of the active components.

### 2.4 Prediction of the Effects of the Volatile Components of *R. rubescens* on Human Target Genes

SwissTargetPrediction and Batman-TCM tools were used to predict the target genes of the volatile components in *R. rubescens*. After uploading Canonical SMILES and InChI structures of the active components, “homo” was selected as the species and the target genes of the volatile components were predicted; the selection condition probability was >0.1 and the score cutoff was >20.

### 2.5 Target Gene Prediction for Gastric Cancer

We used OMIM, DisGeNET, and MALACARDS tools to retrieve target gene information related to gastric cancer.

### 2.6 Establishment of the Compound-target-disease Regulatory Network and the Regulatory Analysis

Cytoscape 3.7.2 software was used to construct a protein interaction network of active components of *R. rubescens* and gastric cancer-related targets. R language was used to analyze the above target gene information. GO annotation analysis and KEGG pathway analysis were performed.

### 2.7 Biological Verification

#### 2.7.1 Cell Culture and Treatment

The human gastric cancer cell line, MKN-45 (Wanleibio Co., Ltd., Shenyang, China), was cultured in the RMI1640 medium containing 20% fetal bovine serum at 37°C under 5% CO_2_.

MKN-45 cells were cultured to the logarithmic growth stage and divided into six groups. Cells were treated with different doses of *R. rubescens* extract (0, 50, 100, 150, 200, 250 μM) for 48 h before analyses.

#### 2.7.2 CCK-8 Assay

Cells were treated for specific times. The medium was replaced with 100 μL fresh medium and 10 μL CCK-8 was added. Cells were then incubated in a 37°C incubator for 1 h. The OD value at 450 nm was measured on a microplate analyzer, and data analysis was performed.

#### 2.7.3 Evaluation of Apoptosis by Flow Cytometry

Treated cells were collected by centrifugation, and the supernatant was discarded. The cells were washed twice with PBS and resuspended in 500 μl Binding Buffer (Wanleibio Co., Ltd., Shenyang, China). Next, 5 μL Annexin V-FITC (Wanleibio Co., Ltd.) and 10 μL propidium iodide (Wanleibio Co., Ltd.) were added and samples were mixed. Samples were incubated at room temperature away from light for 5-15 min. Analysis by flow cytometry was then performed.

#### 2.7.4 Western Blotting

Cells were lysed, and the protein concentration of samples was quantified using a protein quantification assay kit (Wanleibio Co., Ltd.). Equal amounts of samples (20 μg) were separated by polyacrylamide gel electrophoresis and transferred to a polyvinylidene difluoride membrane (Millipore, Bedford, MA, USA). The membranes were washed in Tris-buffered saline with 0.5% Tween 20 (TBST) for 5 min and then blocked in 5% skimmed milk for 1 h. The membranes were incubated with antibodies against caspase 3,TNFa, IL1B, MMP9 and PTGS2 cleaved caspase 3, BAD, and BCL2 (1:2000; Wanleibio Co., Ltd) overnight at 4°C. The membranes were washed three times with TBST for 5 min each and then incubated for 1 h at 37°C with a rabbit HRP-conjugated anti-mouse antibody (1:1000) (Wanleibio Co.). The membranes were washed three times with TBST for 5 min each time, and protein bands were detected using an electrochemiluminescence (ECL) agent (Wanleibio Co., Ltd.). β-actin was used as an internal control for expression normalization.

### 2.8 Statistical Analysis

SPSS23.0 and GraphPad Prism 9 were used to analyze and plot the experimental data. T was used to compare data between groups. *P*<0.05 indicated statistical significance.

## 3 RESULTS

### 3.1 Detection and Screening of Volatile Components of *R. rubescens*

An ion flow diagram of the active components of *R. rubescens* is shown in Fig. (**1**). Seventy-eight volatile compounds were obtained by comparison with the NIST database, and the CAS number list of the compounds is shown in Table **1**.

### 3.2 Identification of Volatile Components of *R. rubescens*

GC-MS results were analyzed using the TCMSP database, and 13 volatile components of *R. rubescens* were retrieved: palmitic acid, oleic acid, palmitoleic acid, dihydrocarvyl alcohol, peach aldehyde, ribitol, 2,5-octadecanedioic acid, methyl acrylate, isopropyl palmitate, 4-hydroxy-phenylthiol, 9-decanoic acid, 1-heptatriacotanol, 3,5-ditert-butyl phenol, and salicin (Table **2**). Six met the screening criteria of oral absorption and utilization degree OB ≥30: oleic acid, palmitoleic acid, dihydrocarvyl alcohol, peach aldehyde, 4-hydroxyphenylthiol, and 9-decanoic acid. One compound met the screening criteria for compounds of a medicinal nature DL ≥0.18: 1-heptatriacotanol. Oleic acid, 2,5-octadecanedioic acid, methyl acrylate, isopropyl palmitate, and salicin also showed good chemical properties.

### 3.3 Prediction of Targets in Gastric Cancer by Volatile Components of *R. rubescens*

Thirteen compounds were detected in the volatile components of *R. rubescens* using the TCMSP database. Using the SwissTargetPrediction platform and BATMAN-TCM platform, 839 target genes of the 13 volatile components were predicted. Cytoscape 3.7.2 software was used to make a target network diagram of the volatile components of *R. rubescens*, as shown in Fig. (**2**).

**Fig. (3):**
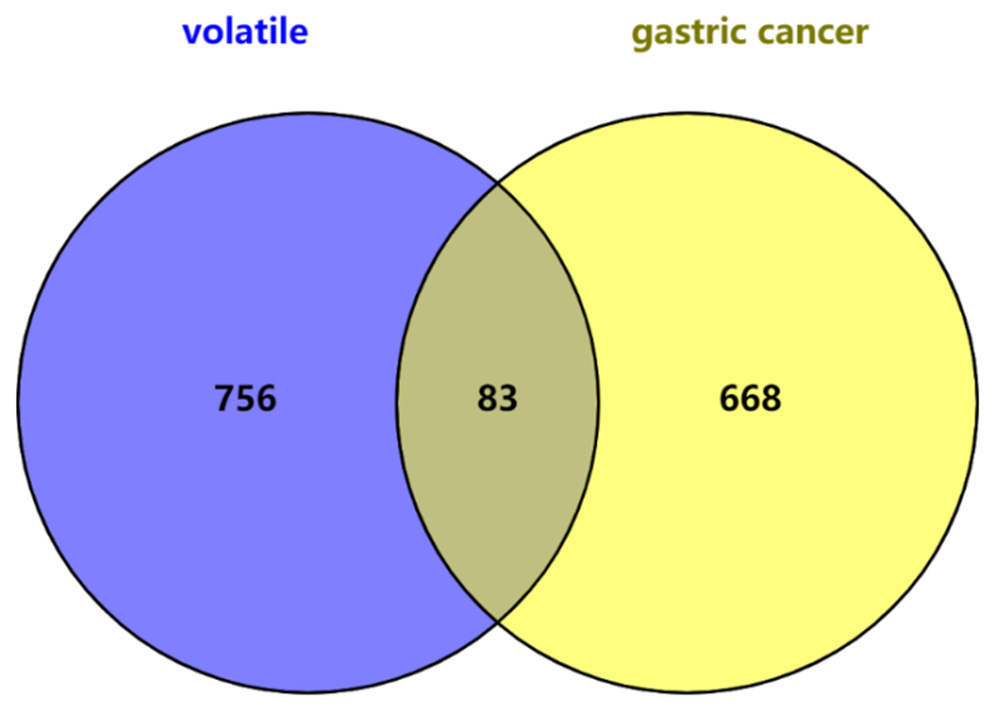
Venn diagram of the targets related to gastric cancer and volatile components of *Rabdosia rubescens*.

Network database screening identified 751 gastric cancer targets and a Venn diagram determined 83 common targets between the volatile components of *R. rubescens* and gastric cancer (Fig. **3**). The list of common targets is shown in Table **3**.

### 3.4 Network Regulation Analysis of Targets related to Volatile Components of *R. rubescens* and Gastric Cancer

The results showed that palmitic acid corresponds to 19 targets, oleic acid corresponds to 9 targets, palmitoleic acid corresponds to 30 targets, dihydrocarvyl alcohol corresponds to 9 targets, peach aldehyde corresponds to 14 targets, ribitol corresponds to 0 targets, 2,5-octadecanedioic acid and methyl acrylate correspond to 6 targets, isopropyl palmitate corresponds to 3 targets, 4-hydroxyphenylthiol corresponds to 0 targets, 9-decanoic acid corresponds to 24 targets, 1-heptatriacotanol corresponds to 10 targets, 3,5-ditert-butyl phenol corresponds to 23 targets, and salicin corresponds to 5 targets. Cytoscape 3.7.2 software was used to construct a target network regulation diagram of the volatile components of *R. rubescens* and gastric cancer, as shown in Fig. (**4**).

Next, R language was used to conduct GO annotation analysis and KEGG pathway enrichment analysis on the 83 common targets; the results are shown in Figs. (**5** and **6**).

Targets were enriched in biological processes, including muscle cell proliferation, reproductive structure development, reproductive system development, steroid hormone response, and epithelial cell proliferation; terms in cellular components were membrane raft, membrane microregion, membrane region, caveola, plasma membrane raft, collagen-containing extracellular matrix, vesicle lumen, and WNT signalosome; terms in molecular function were steroid hormone receptor activity, nuclear receptor activity, transcription factor activity, direct ligand regulatory sequence specific DNA binding, receptor ligand activity, phosphatase binding, protein phosphatase binding, transmembrane receptor protein tyrosine kinase activity, and RNA polymerase three-factor binding.

The main signaling pathways enriched by KEGG included gastric cancer, breast cancer, proteoglycans in cancer, EGFR tyrosine kinase inhibitor resistance, endocrine resistance, hepatocellular carcinoma, MAPK signaling pathway, and hepatitis B.

### 3.5 Core Targets of Volatile Components of *R. rubescens* in Gastric Cancer and Protein-protein Interaction Regulatory Network

The 83 common targets were imported into the STRING database platform to build a protein-protein interaction network. Cytoscape 3.7.2 was used for screening and identified four core targets, TNF, IL1B, MPP9, and PTGS2, as shown in Fig. (**7**).

The volatile components of *R. rubescens* corresponding to the four targets, TNF, IL1B, MMP9, and PTGS2, were inquired through feedback as follows. Four compounds target TNF: oleic acid, palmitoleic acid, 9-decenoic acid, and 3,5-di-tert-butylphenol. Four compounds target IL1B: oleic acid, palmitoleic acid, 9-decenoic acid, and 3,5-di-tert-butylphenol. One compound targets MMP9: peach aldehyde. Four compounds target PTGS2: oleic acid, palmitoleic acid, peach aldehyde, and 9-decenoic acid.

### 3.6 Experimental Verification of the Effects of Volatile oil Components of *R. rubescens* on Gastric Cancer Cells

CCK-8 assays were used to analyze the effects of different concentrations of *R. rubescens* volatile oil on gastric cancer cell viability, and the results are shown in Table **4**. The volatile oil of *R. rubescens* inhibited the viability of gastric cancer cells in a concentration-dependent manner. From these results, we selected 50 μg/mL as the low concentration and 150 μg/mL as the high concentration for subsequent experiments.

**Fig. (8):**
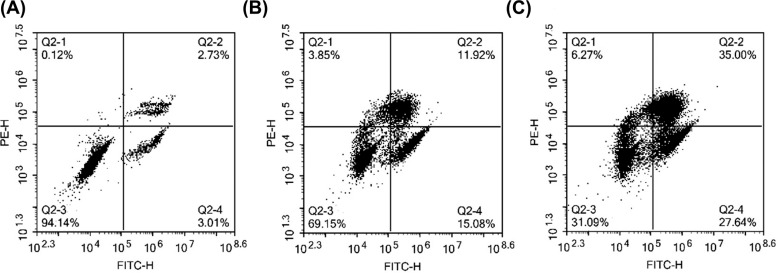
Flow cytometry results. **Note:** (**A**): MKN-45 control group; (**B**): *Rabdosia rubescens* volatile component low-dose group; (**C**): *Rabdosia rubescens* volatile component high-dose group.

To evaluate the influence of the volatile components of *R. rubescens* on the apoptosis of gastric cancer cells, we performed flow cytometry analysis (Fig. **8**) and analyzed the apoptosis rate (Table **5**). The apoptosis rate of the MKN-45 control group was 5.74%, while that of the low-dose group was 27%, and that of the high-dose group was 62.64%. The apoptotic rate of the high-dose volatile oil components of *R. rubescens* was significantly higher than that of the low-dose group. These results indicate that the high concentration of *R. rubescens* volatile components promoted the apoptosis of gastric cancer cells.

**Fig. (9):**
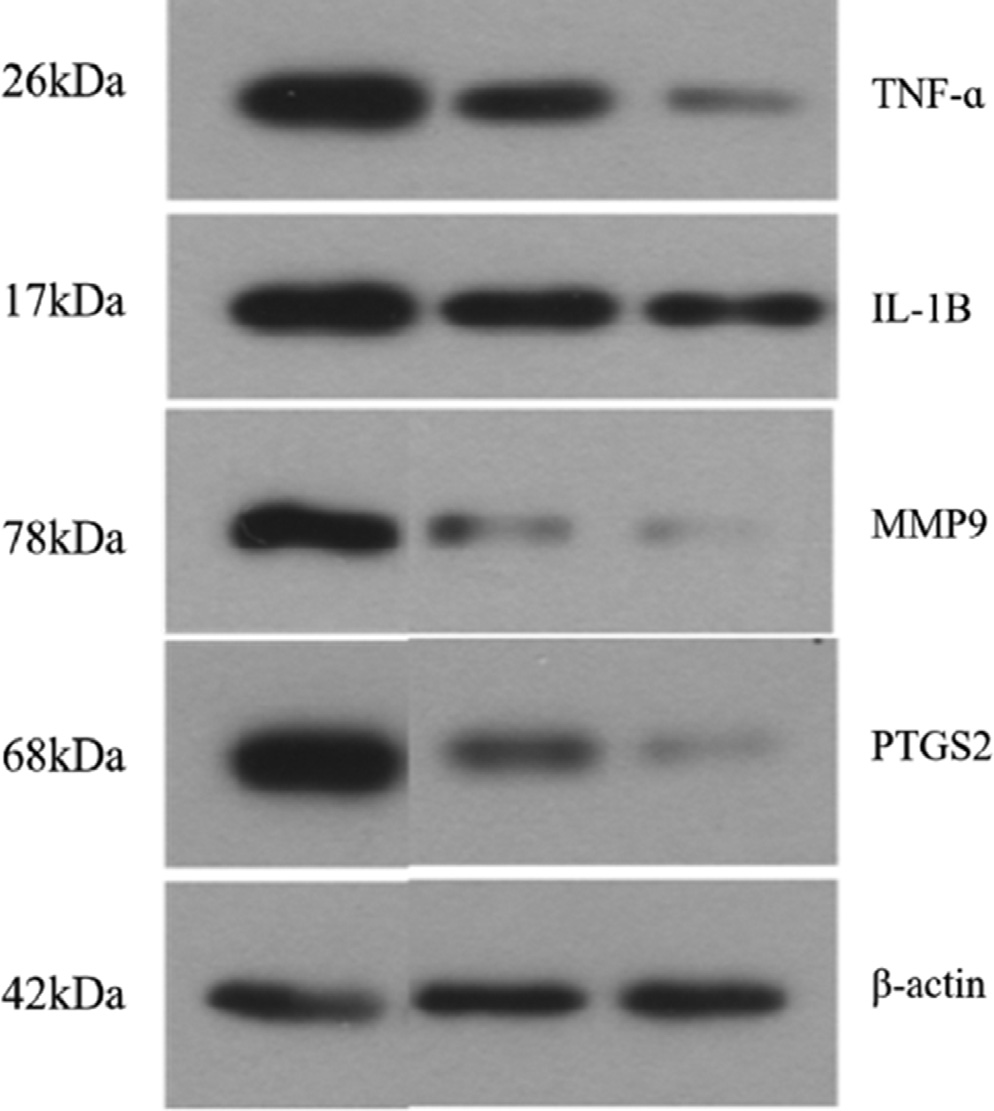
Western blot analysis.

**Fig. (10A-D):**
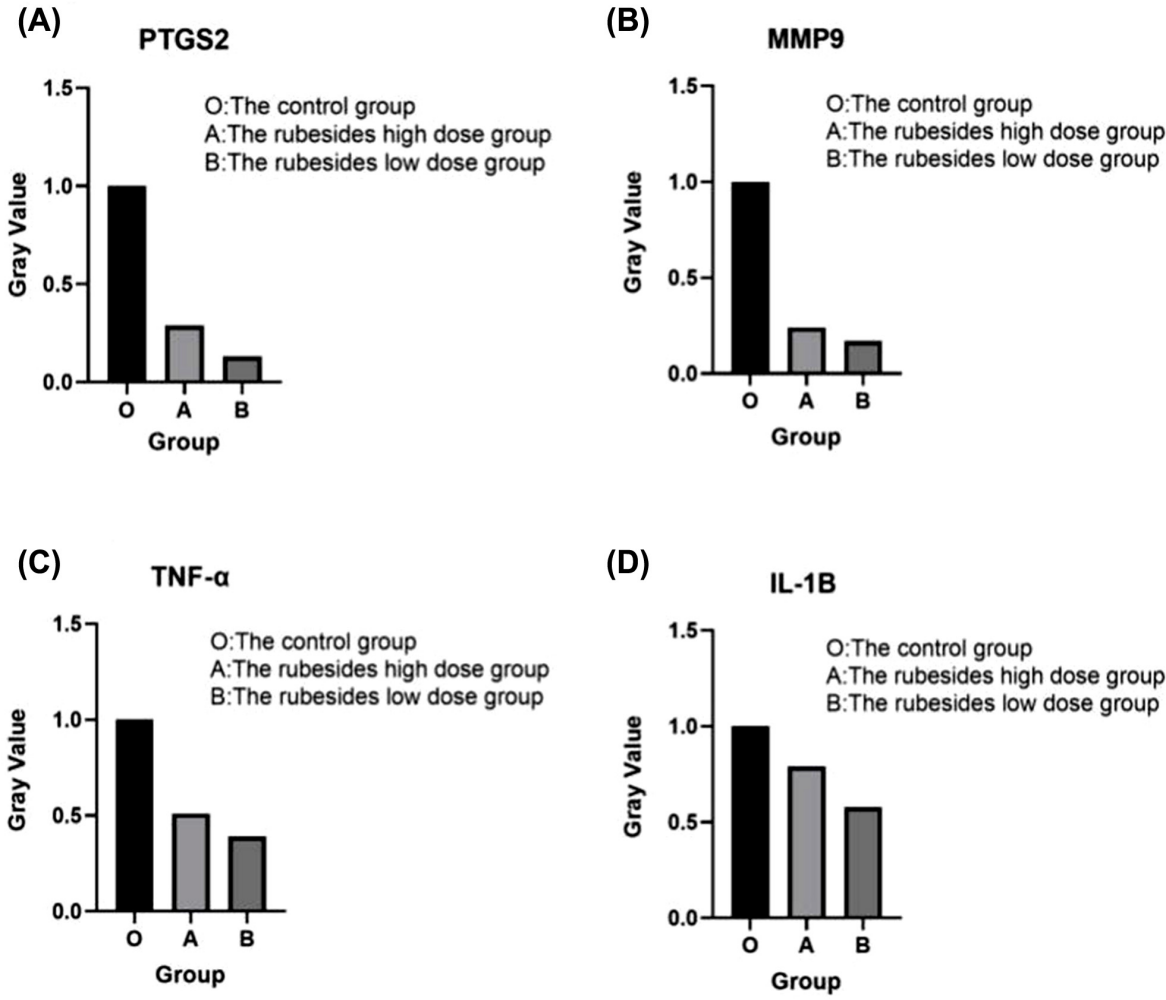
Gray value analysis O: MKN-45 untreated group: *Rabdosia rubescens* volatile component high-dose group; *Rabdosia rubescens* volatile component low-dose group.

To further examine the anti-apoptosis mechanism of the volatile oil of *R. rubescens* in gastric cancer cells, we performed western blotting to detect the four core targets, TNF, IL1B, MPP9 and PTGS2. The results showed that levels of TNF, IL1B, MPP9, and PTGS2 in the low-dose group were significantly higher than those of the high-dose group (Figs. **9** and **10**). These results show that the high concentration of *R. rubescens* volatile oil can effectively reduce the levels of TNF, IL1B, MPP9, and PTGS2.

## 4 DISCUSSION

*R. rubescens* has excellent anti-tumor effects against a variety of cancers [9]. *R. rubescens* not only greatly improves the anti-tumor effect of chemotherapy drugs but also reduces the required dosage and side effects [10, 11]. *R. rubescens* contains a large amount of volatile oil, mostly monoterpenes and long-chain hydrocarbons, including α-pinene and β-pinene, limonene, 1,8-eucalyptusp-paragenin, nonanal, decanal, β-elemene, palmitic acid, tridecane, and squalene [12]. Limonene, α-pinene, β-pinene, β-elemene, and other volatile components have anti-tumor effects [13, 14]. Luting *et al*. [15] showed that β-elemene induces protective autophagy in SGC-7901 cells, and downregulation of the Beclin1 gene reduces the occurrence of protective autophagy, thus enhancing the anti-cancer effect of β-elemene. Yang *et al*. [16] demonstrated that β-elemene inhibits the proliferation of SGC7901/ADR cells in a concentration-dependent manner. Another study showed that β-elemene inhibits the invasion and metastasis of multidrug resistant gastric cancer SGC7901/ADR cells and drug resistance and metastasis induced by SGC7901/ADR cell exosomes [17]. In addition, *in vitro* experiments showed that α-pinene, β-pinene, and limonene significantly inhibit the proliferation and migration of MGC-803 gastric cancer cells, inhibit the G2/M phase of the cell cycle, decrease the mitochondrial membrane potential, and induce apoptosis. α-pinene, β-pinene, and limonene may also play an anti-cancer role in gastric cancer through the HIPPO/YAP signaling pathway [18]. The effects of limonene and pinene on gastric cancer have been reviewed [19].

In this paper, GC-MS detection combined with the TCMSP database for ADME analysis identified 13 volatile functional components of *R. rubescens*. Using the SwissTargetPrediction platform and the BATMAN-TCM platform, 751 human targets were predicted, and 83 common targets were obtained by matching with gastric cancer disease gene targets. Four core targets were identified by Cytoscape 3.7.2 software: TNF, IL1B, MMP9 and PTGS2.

Western blotting showed that the levels of TNF, IL1B, MPP9, and PTGS2 were significantly higher in the low-dose group compared with the high-dose group. These results showed that a high concentration of *R. rubescens* volatile oil can effectively reduce the levels of TNF, IL1B, MPP9, and PTGS2. The apoptosis rate of the high-dose *R. rubescens* volatile oil group was significantly higher than that of the low-dose group, indicating that high concentrations of *R. rubescens* volatile oil can effectively promote the apoptosis of gastric cancer cells. Our findings indicate that the volatile oil of *R. rubescens* may promote the apoptosis of gastric cancer cells by inhibiting the expression of TNF, IL1B, MPP9, and PTGS2.

TNF, IL1B, and MMP9 play important roles in the occurrence and development of gastric cancer. To our knowledge, no studies have examined the role of PTGS2 in gastric cancer. TNF is an important inflammatory factor with various activities and allelic polymorphisms of TNF-A-308, TNF-B-1069, and TNF-B-252A/G, and other loci are associated with the incidence and susceptibility of gastric cancer [20, 21]. Lin [22] proposed that the genetic polymorphisms of IL1B-511 and IL1B-31 in a Han Chinese population were associated with susceptibility to gastric cancer, and clinical tests showed that the T type of IL1B-511 or the C type of IL1B-31 increased the incidence rate of gastric cancer. Liang *et al*. [23] found that TNFa and IL1B promoted adhesion between gastric cancer cells and peritoneal mesothelial cells by increasing the levels of ICAM1, VCAM1 and CD44 mRNAs, which play a positive role in the peritoneal implantation and metastasis of gastric cancer cells.

KEGG enrichment analysis showed that the target genes may be involved in gastric cancer through effects on the MAPK signaling pathway, proteoglycan in cancer, EGFR tyrosine kinase inhibitor resistance, and other biological pathways. The MAPK pathway plays multiple roles in cellular processes, and the mitogen-activated protein kinase in the pathway participates in cell migration and infiltration by regulating the expression and activation of MMPs and FAK [24-26]. Yajun *et al*. showed that activation of MAPK and NF-κB led to phosphorylation of IKB, which leads to the transcriptional upregulation of apoptotic genes and eventually results in activation of the effector caspase 3, nuclear DNA breakdown, and complete disintegration of the cell structure, leading to cell death [27]. EGFR is a receptor glycoprotein that regulates the DNA repair response and reduces DNA repair ability by interacting with factors related to the DNA repair system. The EGFR/p38 signaling pathway is involved in the occurrence and development of a variety of tumors and plays an important regulatory role in tumor cell proliferation, apoptosis, invasion, and metastasis. Most gastric cancers exhibit abnormal activation of this pathway. Over-activation of the EGFR/p38 signaling pathway promotes cell proliferation, inhibits apoptosis, and promotes cell invasion [28]. Shang *et al*. [29] showed that the TCM compound, Babaodan, induces the apoptosis of gastric cancer cells through MAPK and NF-κB signaling pathways. Deng *et al*. used β-elemene to inhibit the metastasis of MDR gastric cancer cells through the miR-1323/CBL-b/EGFR pathway, and β-elemene significantly inhibited the metastasis of MDR gastric cancer cells *in vitro* and *in vivo*. Deng *et al*. [30] showed that β-elemene upregulates the expression of CBL-B, thereby inhibiting the EGFR-ERK/Akt pathway, regulating the expression of MMP2/9 and reversing epithelial-mesenchymal transformation.

TNF, IL1B, and MMP9, the targets of volatile compounds in *R. rubescens*, are critical factors in MAPK and NF-κB signaling and in the EGFR/p38 signaling pathway [31-33]; therefore, we speculate that volatile components of *R. rubescens* may be useful for the treatment and prevention of gastric cancer through targeting TNF, IL1B and MMP9. Our findings indicate that *R. rubescens* may have therapeutic effects on gastric cancer and other tumors. However, the anti-tumor studies of volatile components of *R. rubescens* for gastric cancer and other tumors are not comprehensive; therefore, in-depth *in vivo* and *in vitro* experiments are still needed to confirm their effects.

## CONCLUSION

This study adopted a network pharmacology method to analyze the key targets and mechanisms of volatile components of *R. rubescens* on gastric cancer. We found that the volatile components of *R. rubescens* could inhibit the activity of gastric cancer cells, possibly by regulating the levels of TNF, IL1B, MPP9 and PTGS2. Our study provides new theoretical support for volatile components of TCM in the treatment of gastric cancer and other tumors.

## Figures and Tables

**Fig. (1):**
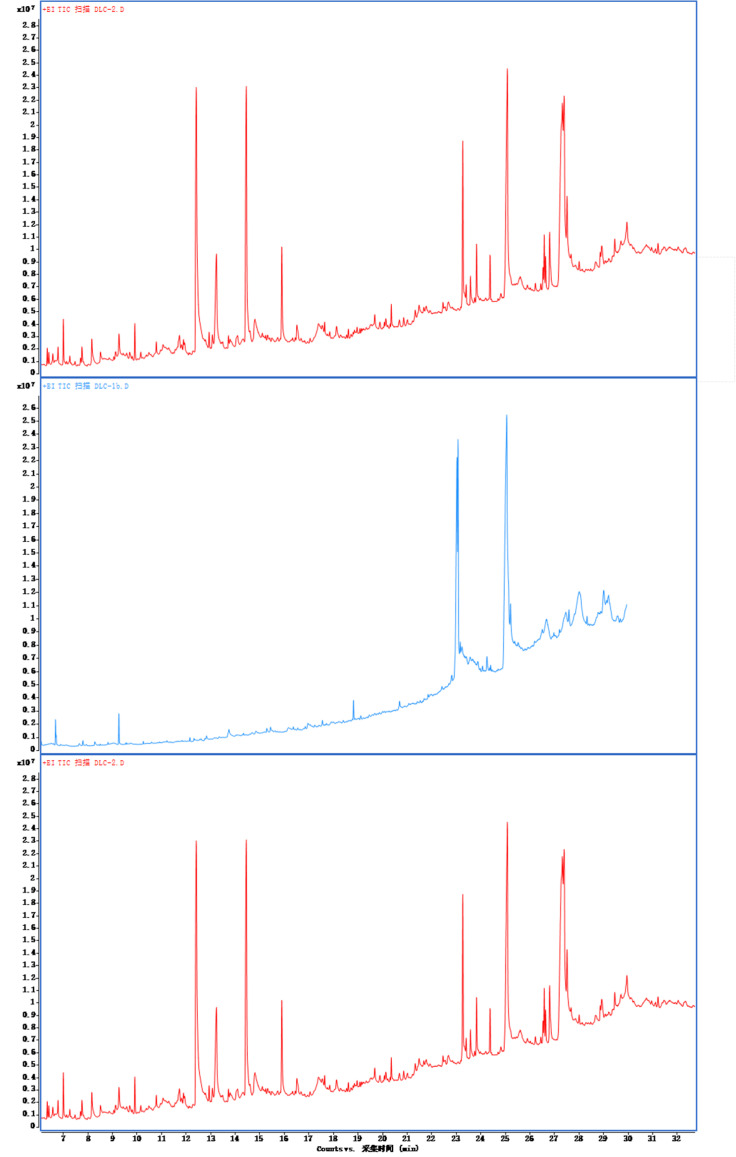
Ion flow diagram for the detection of *Rabdosia rubescens* extract (methanol, ethanol, benzenol) by GC-MS.

**Fig. (2):**
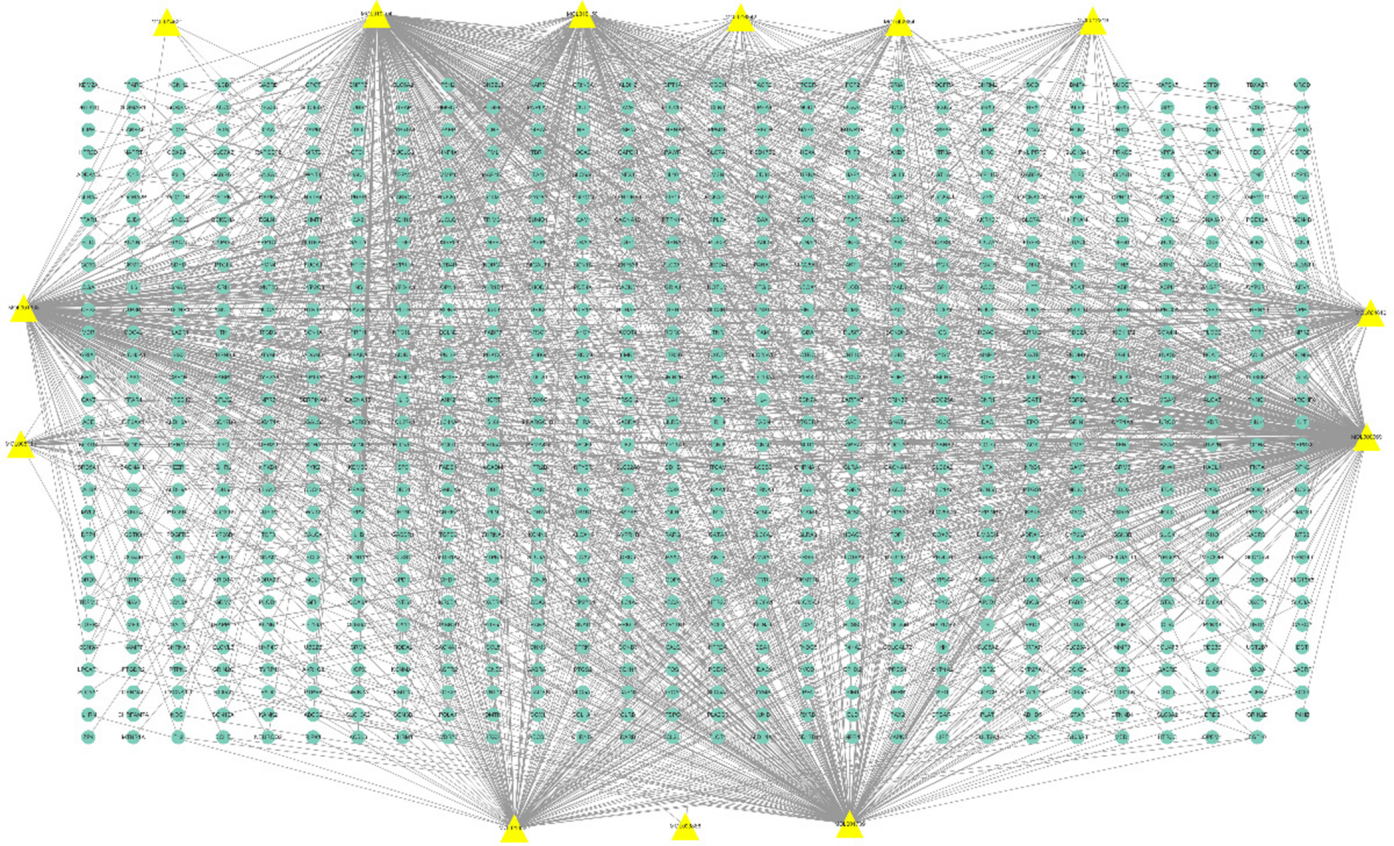
Target network of volatile components in *Rabdosia rubescens*.

**Fig. (4):**
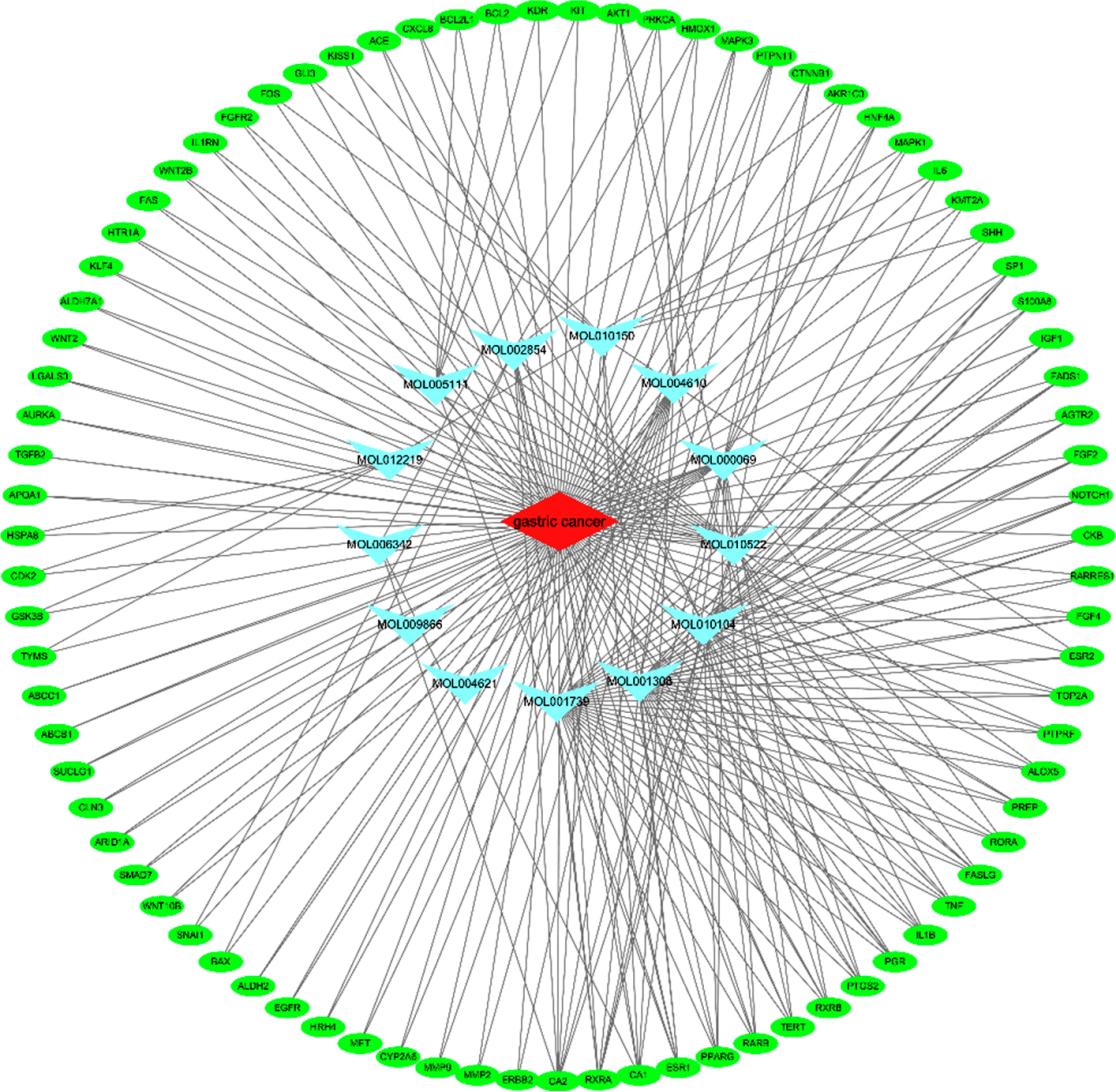
Regulatory network of volatile constituents in *Rabdosia rubescens*-gastric cancer targets.

**Fig. (5):**
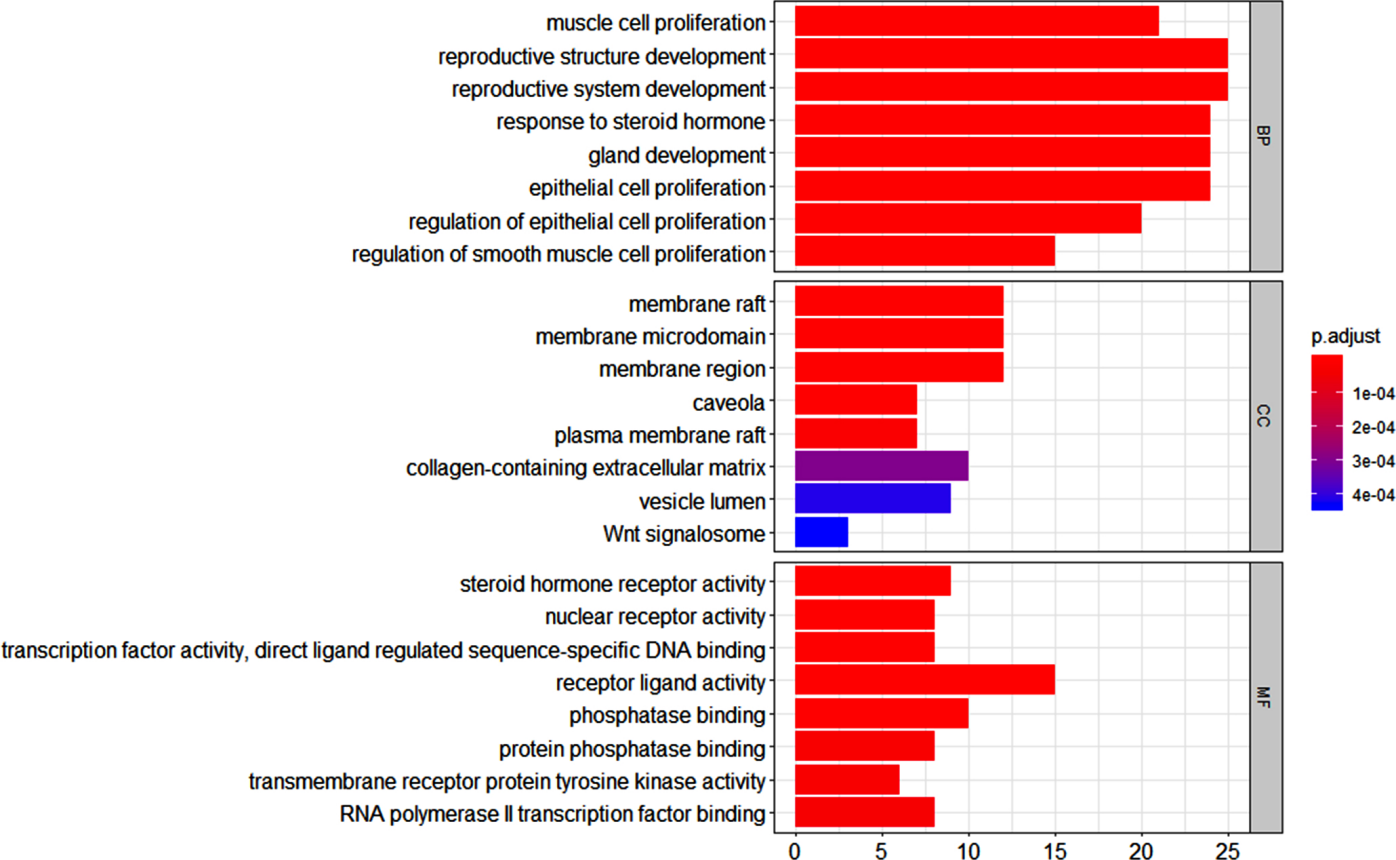
GO annotation of the targets related to gastric cancer and volatile components of *Rabdosia rubescens.*

**Fig. (6):**
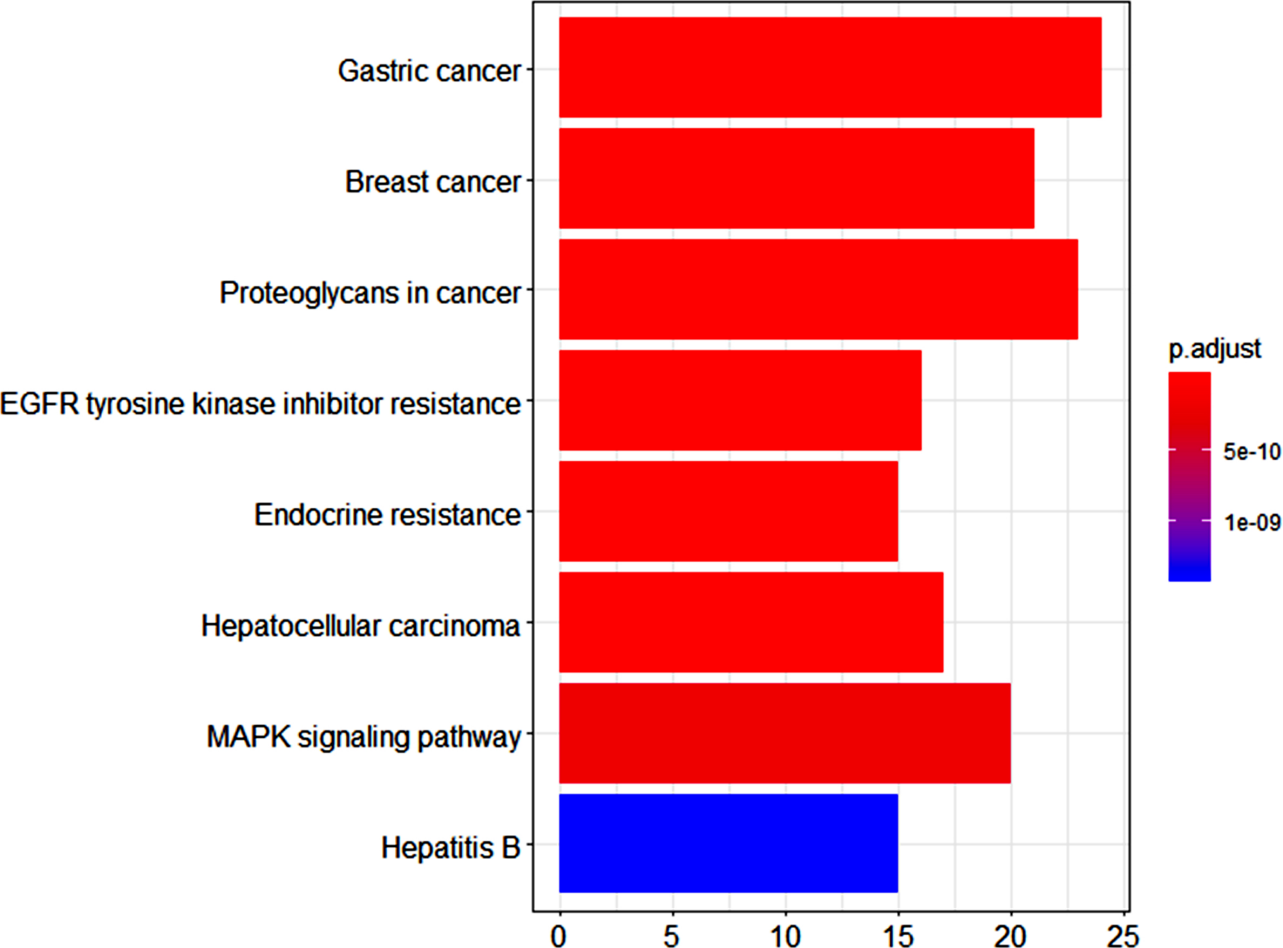
KEGG pathway analysis of the targets related to gastric cancer and volatile components of *Rabdosia rubescens*.

**Fig. (7):**
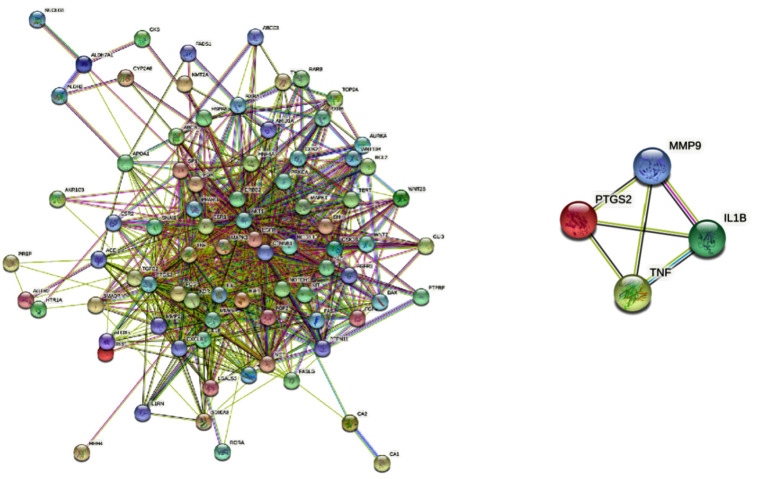
Protein-protein interaction network and core targets of the volatile components of *Rabdosia rubescens* acting on gastric cancer.

**Table 1:** List of 78 volatile components in *Rabdosia rubescens* extract.

**CAS Number List of Volatile Components of *Rabdosia rubescens***				
1000043-05-3	1825-00-9	1000374-07-3	55947-04-1	1000357-87-0
1000125-84-4	2177-18-6	1000374-63-1	56051-53-7	1000367-91-1
1000130-97-4	2777-66-4	1000443-09-1	56528-77-9	1679-53-4
1000130-99-6	33535-07-8	10230-62-3	56554-30-4	55724-48-6
1000185-23-1	3444-72-2	104-67-6	56846-98-1	55759-94-9
1000187-50-7	35034-15-2	105794-58-9	57-10-3	91875-71-7
1000194-28-5	35810-56-1	1124-15-8	57156-91-9	1551-24-2
1000212-02-6	373-49-9	112-80-1	597-12-6	16654-42-5
1000282-18-2	38049-26-2	1138-52-9	621-58-9	16725-41-0
1000283-75-5	4208-60-0	118068-76-1	637-89-8	77508-64-6
1000300-56-5	443-82-3	128-13-2	6919-52-4	77573-35-4
1000313-37-7	4727-18-8	130403-61-1	71-43-2	1978-10-4
1000333-05-1	4766-57-8	13126-39-1	7459-33-8	77171-29-0
1000336-33-7	488-81-3	138-52-3	74602-32-7	14436-32-9
1000336-66-8	5115-81-1	142-91-6	7681-79-0	5485-65-4
1726-77-8	98183-35-8	1000356-78-4	-	-

**Table 2:** Identification of volatile components in *Rabdosia rubescens*.

**Molecule Name**	**CAS**	**Molecule ID**	**OB (%)**	**DL**
Palmitic acid	57-10-3	MOL000069	19.3	0.1
Oleic acid	112-80-1	MOL001308	33.13	0.14
Palmitoleic acid	373-49-9	MOL001739	35.78	0.1
Dihydrocarvyl alcohol	38049-26-2	MOL002854	51.17	0.03
Peach aldehyde	104-67-6	MOL004610	49.12	0.04
Ribitol	488-81-3	MOL004621	17.61	0.02
2,5-octadecanedioic acid, methyl acrylate	57156-91-9	MOL005111	6.88	0.17
Isopropyl palmitate	142-91-6	MOL006342	19.51	0.16
4-hydroxyphenylthiol	637-89-8	MOL009866	60.34	0.01
9-decanoic acid	14436-32-9	MOL010104	45.65	0.03
1-Heptatriacotanol	105794-58-9	MOL010150	9.83	0.39
3,5-Ditert-butyl phenol	1138-52-9	MOL010522	19.95	0.06
Salicin	138-52-3	MOL012219	7.15	0.16

**Table 3:** List of volatile components of *Rabdosia rubescens* common with target genes in gastric cancer.

**83 Common Targets for Gastric Cancer and Volatile Components of *R. rubescens***						
CA2	ALOX5	S100A8	ACE	CDK2	SUCLG1	IL1B
CA1	PTPRF	SP1	SHH	GSK3B	CTNNB1	AGTR2
PPARG	PTGS2	HMOX1	KISS1	TYMS	CLN3	FADS1
RXRA	PTPN11	SNAI1	KMT2A	WNT10B	MMP2	ALDH7A1
RARB	MAPK3	BAX	GLI3	PREP	ERBB2	WNT2
TERT	TOP2A	PRKCA	FOS	IGF1	KIT	LGALS3
RXRB	PGR	ALDH2	IL6	FASLG	BCL2	AKR1C3
ABCC1	ESR2	EGFR	FGFR2	BCL2L1	TGFB2	ARID1A
ABCB1	FGF4	HRH4	IL1RN	CXCL8	AURKA	TNF
MAPK1	RARRES1	AKT1	WNT2B	APOA1	FGF2	MMP9
RORA	CKB	MET	FAS	HSPA8	KLF4	ESR1
HNF4A	NOTCH1	CYP2A6	HTR1A	SMAD7	KDR	-

**Table 4:** The effects of different concentrations of volatile oil components of *Rabdosia rubescens* on cell viability.

**MKN-45**	**Absorbance (450 nm)**	**Inhibition Rate (%)**
A:MKN-45 control group	0.783±0.040	0.00
B:50 μg/mL *Rabdosia rubescens* volatile oil component group	0.560±0.029	28.41
C:75 μg/mL *Rabdosia rubescens* volatile oil component group	0.488±0.016	37.66
D:100 μg/mL *Rabdosia rubescens* volatile oil component group	0.416±0.026	46.88
E:125 μg/mL *Rabdosia rubescens* volatile oil component group	0.341±0.016	56.49
F:150 μg/mL *Rabdosia rubescens* volatile oil component group	0.296±0.021	62.21

**Table 5:** Evaluation of apoptosis by flow cytometry.

**MKN-45**	**Apoptosis Rate (%)**
A:MKN-45 normal group	5.74
B: *Rabdosia rubescens* volatile oil component low-dose group	27.00
C: *Rabdosia rubescens* volatile components high-dose group	62.64

## Data Availability

The datasets generated and/or analyzed during the current study are available from the corresponding author [Y.H] upon reasonable request.
